# Getting Older People’s Voices Heard: A Quantitative Study Using the Validated Italian Age-Friendly Cities and Communities Questionnaire in Venice, Verona and Palermo

**DOI:** 10.1007/s10823-025-09532-1

**Published:** 2025-05-14

**Authors:** Michele Bertani, Joost van Hoof, Jeroen Dikken

**Affiliations:** 1 Department of Economics, Ca’ Foscari University of Venice, Cannaregio, 873, Venezia, 30100 Italy; 2 Research Group of Urban Ageing, Faculty of Social Work & Education, The Hague University of Applied Sciences, Johanna Westerdijkplein 75, Den Haag, 2521 EN The Netherlands; 3 Department of Systems Research, Faculty of Spatial Management and Landscape Architecture, Wrocław University of Environmental and Life Sciences, ul. Grunwaldzka 55, Wrocław, 50-357 Poland

**Keywords:** Ageing, Social policies, Citizen engagement, Age-friendly community, Italy, AFCCQ-IT

## Abstract

The convergence of population ageing with urbanisation is one of the key challenges to European societies. Italy is at the forefront of this trend, as 24.1% of its population is aged 65 or above, and nearly 72% of its total population lives in urban areas. Given Italy’s demographic scenario, it is important to design age-friendly policies on the national and local levels. The paper contributes to the research of this topic through a technique for analysing citywide assessment data on age-friendliness. Our study presents the process of validation for the Italian older population of a standardised tool: the Age-Friendly Cities and Communities Questionnaire. The study was conducted in three Italian cities (*n* = 1,213) on a representative sample of older people who were asked to rate their life in the city, following the dimensions considered essential for a community’s age-friendliness by the World Health Organization. Four typologies were differentiated in the analysis, resulting in different views of older adults on their experience of living in the city, showing that people’s socioeconomic status and their health situation play a role in shaping their perceptions of age-friendliness. The results highlight the importance of standardised tools for effective social policies for age-friendly cities.

**Supplementary Information:**

The online version contains supplementary material (The Age-Friendly Cities and Communities Questionnaire, Italian version) available at 10.1007/s10823-025-09532-1.

## Introduction

Population ageing stands out as a significant and unprecedented phenomenon of the 21 st century. This trend has far-reaching implications worldwide, impacting the increased likelihood for individuals to attain old age, albeit not always in optimal health. Additionally, there is a notable ascent in the proportion of older individuals relative to the total population (Edmonston, 2021; Khan, 2019; Mahmood & Dhakal, 2023).

According to the United Nations, more than half of the global population resides in urban areas (56.5%), which is anticipated to rise to approximately 68% by 2050 (United Nations, 2022a, 2022b). Regions worldwide have experienced diverse urbanisation patterns, with North America and Europe currently standing out as the most urbanised areas (with over 75% of the population residing in urban communities).

In line with previously expected long-term trends, the European Union’s population is projected to continue ageing and shrink significantly over the coming generations (European Central Bank, 2023).

Italy is at the forefront of these global ageing trends, scoring the highest percentage in Europe of older adults and the second worldwide after Japan: with a total population of nearly 59 million, around 14.5 million (24.3%) are aged 65 or above. About half of them are aged 74 years or above, and 4.5 million are 80 years or above. (United Nations Population Division, 2022). Italy’s life expectancy ranks among the highest globally, standing at 83.4 years at birth in 2024 (ISTAT Italian Institute for Statistics, 2025).

The convergence of these two global trends – the escalating pace of urbanisation and the ageing of societies – has spurred the World Health Organization’s (WHO) initiative on age-friendly cities and communities (WHO, 2007a, 2007b, 2015, 2023; van Hoof et al., 2021). This movement advocates for solutions to enable older individuals to age actively and enjoy their later years by enhancing their well-being and promoting socio-economic participation. As Greenfield (2018: 41) argues, discourse on communities and ageing traditionally has focused on the availability, accessibility, and quality of local services to support older individuals needing assistance. However, in recent times, a growing worldwide ‘age-friendly’ movement has expanded the conceptualisation of community support for an ageing society beyond mere service delivery. The term ‘age-friendly’ is used in considering how various aspects of a community facilitate or hinder the health and well-being of individuals as they experience long lives. Frameworks on age-friendliness include attention to health and community services for older adults but also encompass other aspects of communities, such as the physical design of outdoor spaces and buildings. Moreover, the WHO (2022a, 2022b, 2022c) emphasizes the importance of citizen engagement as a participatory process which includes the voices of citizens in policy-making increasing public interest in, and understanding of, evidence and political processes, which in turn enhances the legitimacy of policy decisions as well as societal trust. However, one of the critical aspects emerging from the ongoing debate is the multitude of methodological approaches adopted for measuring the age-friendliness of a city or community and the deep differences across sociopolitical landscapes, both between and within countries and continents (Greenfield & Buffel, 2022: 5–6). For instance, Black and Oh (2022) employed a qualitative research method encompassing the analysis of formal reports using content analysis to assess the age-friendly progress reported by American communities. In Hong Kong SAR, Wong et al. (2015) developed a survey to compare the age-friendliness of different neighbourhoods and Chui et al. (2022) implemented a mixed-methods concurrent parallel research design – questionnaires and focus groups – to examine changes over time in perceived age-friendliness among community-dwelling older adults. For Italy, two recent works have analysed different aspects. The first is a study for the measurement of the city age-friendliness of Macerata (Monachesi, 2023), located in the Marche region, adapting to the local context of the survey designed for Hong Kong SAR by Wong et al. (2015) and the second is more oriented to urbanistic and architectural aspects. Luciano et al. (2023) developed the Italian Age-friendly House Scorecard (IAHS), a tool that allows housing owners and designers to assess the age-friendliness of existing housing environments. Other qualitative and quantitative methods to assess the age-friendliness of cities and communities have been summarised by van Hoof et al. (2021).

In Europe, a multitude of urban communities align with the age-friendly agenda, actively participating as members of the network promoted by the WHO. However, still there is a lack of reliable and standardised empirical tools for measuring the cities’ age-friendliness. A proposal to respond to this important challenge is the Age-Friendly Cities and Community Questionnaire (AFCCQ), developed in the Netherlands by Dikken et al. (2020), a tool for measuring age-friendliness, following the criteria of the COnsensus-based Standards for the selection of health Measurement Instruments (Mokkink et al., 2010). The instrument has been used to study the age-friendliness of the city of The Hague over time (van Hoof et al., 2022; Hoof et al., 2024), one of the members of the Global Network for Age-Friendly Cities and Communities.

This article presents the process of translation and validation for the Italian older population of the AFCCQ (Dikken et al., 2020), based on a representative sample of older people, who were asked to rate their perception of the age-friendliness of the city, following specific dimensions related to the quality of life in the urban context. Questionnaires were collected in three Italian cities – Venice, Verona and Palermo – and the perspectives on age-friendliness were analysed accordingly.

## Materials and Methods

This study followed a cross-sectional design, using the Age-Friendly Cities and Community Questionnaire (AFCCQ) (Dikken et al., 2020) as a singular self-assessment tool administered at a specific moment in time. The goal was to assess the perceptions of older adults living in three cities across Italy (Verona, Venice and Palermo) regarding the age-friendliness of their cities and neighbourhoods. Responses are recorded on a 5-point Likert scale, with higher scores indicating a more age-friendly city.

**Fig. 1:**
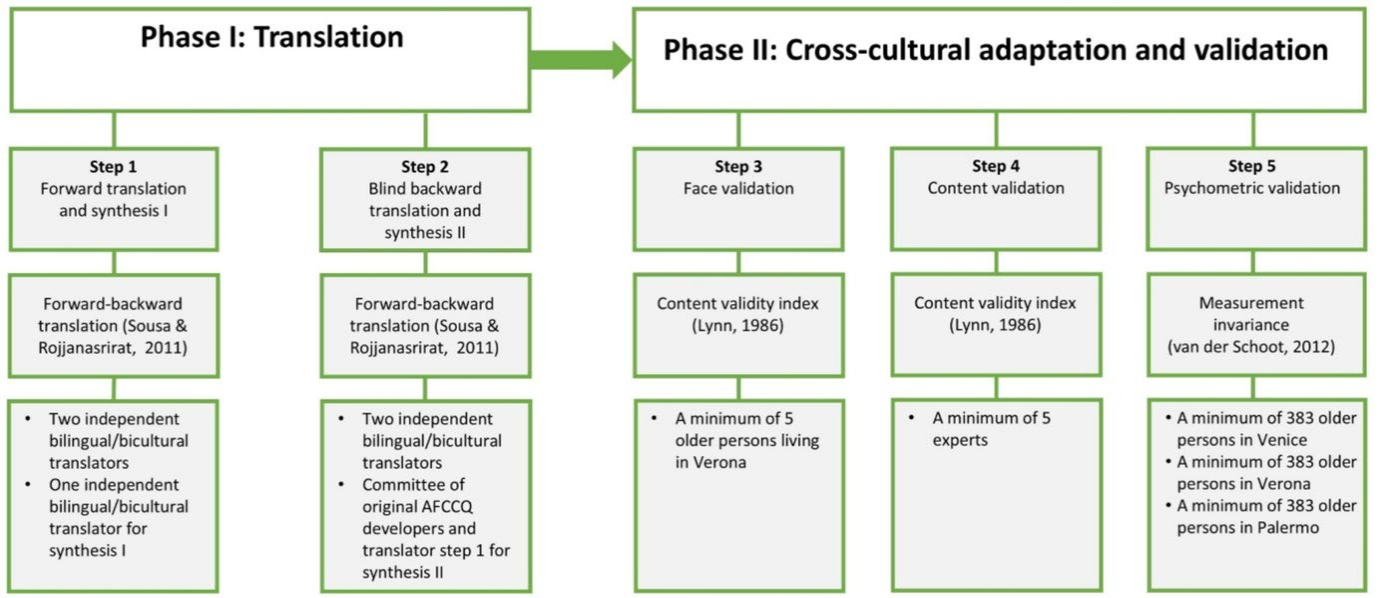
Translation, adaptation, and validation process of the AFCCQ for Italy

The AFCCQ has been adapted and cross-culturally validated previously for the Netherlands (Dikken et al., 2020), Turkey (Özer et al., 2022), Japan (Yamada et al., 2023), Israel (Ayalon et al., 2024), North Macedonia (Pavlovski et al., 2024), Romania (Ivan et al., 2024), Poland (Perek-Białas et al., 2024), Australia (Wasserman et al., 2025), and Russia (Ziganshina et al., 2025). The AFCCQ underwent a comprehensive translation and validation process to ensure the validity of results. This involved multiple steps to ensure rigour, including forward-backward translation, qualitative cultural adaptation assessment with face and content validity using the methods proposed by Lynn (1986) and Polit et al. (2007), followed by psychometric validation through Confirmatory Factor Analysis (CFA). Additionally, in order to facilitate meaningful comparisons, measurement invariance was assessed across the three cities. Figure 1illustrates the translation, adaptation, and validation process of the AFCCQ for Italy.

### Translation and Validation of the AFCCQ-IT

The original AFCCQ-EN in British English was forward-translated to Italian by two independent translators, who were fluent in both English and Italian. Translator #1, with expertise in Italian social gerontology and policy terminology, and Translator #2, familiar with colloquial phrases, jargon, and idiomatic expressions, worked on capturing the socio-cultural nuances. The procedure was based on the guidelines by Sousa and Rojjanasrirat (2011). The forward-translated versions were compared and refined by the research team. Back-translations into English were conducted by two other independent translators, who produced back-translated versions without any prior knowledge of the AFCCQ-EN. The research team compared the instructions, items, and response templates of the back-translations to ensure accuracy.

Face validation involved five older adults and content validation included five academic experts from Italy. Item Content Validity Index (I-CVI) (a quantification method for this qualitative appraisal) was used, with participants rating item relevance on a four-point Likert scale (1 = not relevant, 2 = somewhat relevant, 3 = quite relevant, 4 = highly relevant). For both groups, the I-CVI was calculated using the following equation:

1 $$\:I-CVI=\frac{NR}{N}$$

where *NR* = number of people rating (3) or (4) and *N* = total number of people. I-CVI values greater than 0.78 were deemed acceptable. Both groups were asked if any themes or items relevant to measuring age-friendliness were missing. Data analysis was done using the software package SPSS version 29.0.

After data collection and screening, a CFA was conducted in which the Italian data were compared with the theoretical model outlined by Dikken et al. (2020). Using CFA, the construct validity of the AFCCQ-IT was tested by determining whether the data aligned with the predefined factor structure of the questionnaire. During the analysis, the variance of each latent factor was standardised to unity to facilitate interpretability and allow for factors to co-vary, ensuring that the relationships between different dimensions of age-friendliness (for instance, latent factors such as Housing, Social Participation, and Respect and Social Inclusion) could be captured accurately. To evaluate the goodness of fit between the Italian data and the proposed model, several fit indices were considered, including normed χ^2^, with values up to 5 indicating adequate fit. The Comparative Fit Index (CFI) and Tucker Lewis Index (TLI) should exceed 0.9, while the Root-Mean Squared Residual (SRMR) should be below 0.08 and the Root-Mean Square Error of Approximation (RMSEA) below 0.08 for a moderate fit.

Finally, we evaluated Measurement Invariance (MI) between the three cities to determine if valid comparisons between the cities were feasible. Only when a certain level of MI has been confirmed, a consistent interpretation of individual items and the underlying latent factor across all participant groups is permitted. In other words, one is allowed to make meaningful comparisons of age-friendliness scores between the cities. For instance, the latent factor Social Participation, which includes items like “I have opportunities to attend social events” and “I feel part of my community”; after the confirmation of MI, the same item scores would indicate a similar level of perceived social participation, regardless of whether the respondent is from Venice, Verona, or Palermo. This process ensures that observed differences in age-friendliness scores truly reflect variations in respondents’ perceptions rather than inconsistencies in how items or latent factors are understood across cities. By confirming MI, the analysis provides a robust foundation for valid cross-city comparisons, enabling policymakers to identify areas of strength and areas requiring targeted improvements in age-friendliness.

Configural invariance was first tested by repeating the CFA procedure, but this time separately for each city independently, ensuring the theoretical operationalisation by Dikken et al. (2020) is valid not only for the whole sample, but for each of the cities included in this study. Subsequently, a series of increasingly constrained models were tested. Metric invariance examined equal factor loadings, while intercepts were allowed to differ. Scalar invariance, indicating comparable construct meaning and item levels, was assessed by constraining both loadings and intercepts to be equal. Finally, the Residual Variance was tested by examining whether the variability in the observed variables that is not accounted for by the latent construct is equivalent across groups. For these analyses, the same model fit indices were used as described by the overall CFA.

Subsequently, the final model’s internal consistency was evaluated using composite reliability, preferred over Cronbach’s alpha in the context of CFA, with a value of 0.70 considered appropriate for reliability. These analyses were conducted using IBM SPSS Amos version 29.0 (IBM Corp., 2022).

### Recruitment and Participants

This study was conducted in three Italian cities: Verona, Venice (Italian: *Venezia*), and Palermo. The cities were chosen to design and evaluate two possible comparisons: the first at the intra-regional level with Verona and Venice, located in the same Region (Veneto), and the second at the North vs. South level (Table 1).

**Table 1:**
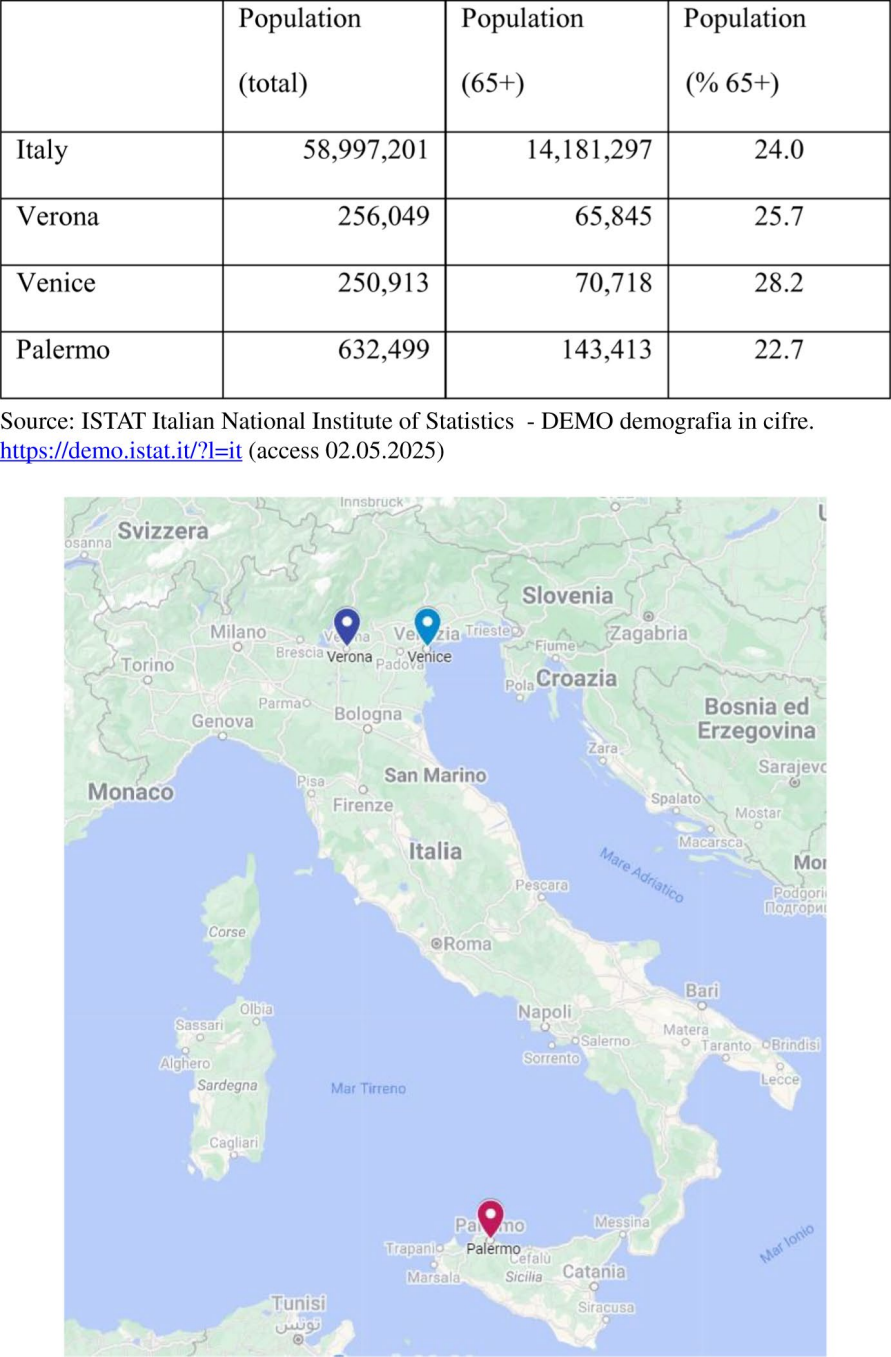
Italy and the municipalities of Verona, Venice and Palermo. Population registered at 01.01.2023

Source: Google. (n.d.). Google Maps of Italy. https://www.google.it/maps/. (Access 02.05.2025)

The cities of Venice and Verona were chosen considering their specific cultural and geographical contexts within the same region, Veneto. The city of Palermo, the capital of the Sicily Region, was selected to compare the eventual regional differences for the AFCCQ scores, considering the well-known gap between North and South regarding the quality of local welfare systems. Besides this, the funding for the data collection did not allow for the expansion of the number of cities involved. This study presents for the first time a convergent research design, data collection and data analysis of the AFCCQ for three different cities.

A representative sample of older people (65 years and over) living in Verona, Venice and Palermo were recruited to participate in this study, based on the demographic data of January 2023. Using a margin of error of 5% and a confidence level of 95%, a minimum of 384 respondents needed to be representative for the city of Palermo, 383 for the city of Venice and 382 for the city of Verona.

Recruitment and participation took place between November and December 2023. There were three inclusion criteria; (i) only those aged 65 years or over, (ii) who lived in their own home (i.e., not residing in institutional care), and (iii) were able to communicate in Italian were included. For the sample to be representative, the number of respondents had to reflect certain demographic characteristics of the older population in the three cities involved in the research. The ratio between males and females had to reflect that of the city, and participants had to come from all municipality districts. The study also recruited people who either lived alone or with a spouse, received care services such as house cleaning or help with personal care, lived with chronic conditions, and used mobility aids (wheeled walker or wheelchair). The fully anonymised database was made thanks to the fundamental support provided by the Coordinators of the Statistical Office of the Municipalities of Venice, Venice and Palermo, which delivered an extract from their archive to the researcher.

Data collection was realised through a simple random sampling approach using the municipal population database by a research agency (Demetra opinioni.net, Venice, Italy). Computer-assisted telephone interviewing (CATI) is the technique adopted by the research agency for the AFCCQ-IT data collection.

### City Results and Age-friendly Typologies for Italy

A total of 1,213 older adults from Verona (*n* = 400), Venice (*n* = 407) and Palermo (*n* = 406), participated in the study, with no missing values on AFCCQ-IT items. Alongside the AFCCQ questionnaire, participants provided demographic information (Table 2).

**Table 2:** Demographics of participants Italy (total *n* = 1,213), Verona (*n* = 400), Venice (*n* = 407), and Palermo (*n* = 406)

	Italy (*n* = 1,213)	Verona (*n* = 400)	Venice (*n* = 407)	Palermo (*n* = 406)
Sex				
Male (%)	489 (40.3%)	161 (40.3%)	162 (39.8%)	166 (40.9%)
Female (%)	724 (59.7%)	239 (59.8%)	245 (60.2%)	240 (59.1%)
Age				
Mean (SD)	76.2 (7.2)	77.0 (7.5)	76.7 (7.2)	75.0 (6.9)
Country of birth				
Born in Italy (%)	1,195 (98.5%)	395 (98.8%)	399 (98.0%)	401 (98.8%)
Educational level				
Primary education; Lower secondary education	421 (34.7%)	154 (38.5%)	178 (43.7%)	89 (21.9%)
Upper secondary education; post-secondary; non-tertiary education	473 (39.0%)	151 (37.8%)	170 (41.8%)	152 (37.4%)
Short-cycle tertiary education; Bachelor or equivalent (Bachelor’s degree)	72 (5.9%)	20 (5.0%)	12 (2.9%)	40 (9.9%)
Master’s degree or equivalent (Master’s degree), Doctoral degree or equivalent (Doctorate)	247 (20.4%)	75 (18.8%)	47 (11.5%)	125 (30.8%)
Type of dwelling				
Owner-occupant	1,088 (89.7%)	360 (90.0%)	373 (91.6%)	355 (87.4%)
Public/Social housing	45 (3.7%)	7 (1.8%)	20 (4.9%)	18 (4.4%)
Rent	80 (6.6%)	33 (8.3%)	14 (3.4%)	33 (8.1%)
Living together with other(s)	842 (69.4%)	275 (68.8%)	286 (70.3%)	281 (69.2%)
Receiving care	447 (36.9%)	151 (37.8%)	130 (31.9%)	166 (40.9%)
from family/relative (voluntary)	201 (16.6%)	69 (17.3%)	80 (19.7%)	52 (12.8%)
from a friend/neighbour (voluntary)	25 (2.1%)	12 (3.0%)	7 (1.7%)	6 (1.5%)
from maid/carer (paid)	264 (21.8%)	80 (20.0%)	63 (15.5%)	121 (29.8%)
from other	9 (0.7%)	5 (1.3%)	3 (0.7%)	1 (0.2%)
Living with one chronic condition	356 (29.3%)	115 (28.7%)	121 (29.7%)	120 (29.6%)
Living with two chronic conditions	117 (23.3%)	27 (6.8%)	45 (11.1%)	45 (11.1%)
Living with three or more chronic conditions	36 (3.1%)	13 (3.4%)	11 (2.7%)	12 (2.9%)
Type of chronic condition				
Ever diagnosed with diabetes	169 (13.9%)	47 (11.8%)	54 (13.3%)	68 (16.7%)
Ever diagnosed with kidney failure	54 (4.5%)	18 (4.5%)	15 (3.7%)	21 (15.2%)
Ever diagnosed with lung diseases (such as chronic bronchitis, emphysema, respiratory failure, bronchial asthma)	102 (8.4%)	30 (7.5%)	38 (9.3%)	34 (8.4%)
Ever diagnosed with heart diseases (such as myocardial infarction	183 (15.1%)	53 (13.3%)	67 (16.5%)	63 (15.5%)
Ever diagnosed with cancer (including leukaemias and lymphomas	175 (14.4%)	56 (14.0%)	65 (16.0%)	54 (13.3%)
Ever diagnosed with liver disease (such as cirrhosis)	30 (2.5%)	11 (2.8%)	8 (2.0%)	11 (2.7%)
Using a wheeled walker or a wheelchair	85 (7.0%)	14 (3.5%)	43 (10.6%)	28 (6.9%)
Score for quality of life (scale 1 lowest − 10 highest) mean (SD)	7.4 (2.0)	7.6 (1.8)	7.3 (2.1)	7.4 (1.9)

Initial analyses involved examining mean scores for the whole sample, the three cities and its districts, and examen potential differences between the cities and or districts. Subsequently, cluster analysis was done to create age-friendly typologies, grouping participants based on the nine domains of the AFCCQ. Likert scale data were normalised, and hierarchical cluster analysis (HCA) with Ward’s method determined the number of clusters. Validation involved splitting the sample by city and repeating cluster analysis. The *k*-means cluster analysis provided clarity, with demographic characteristics examined to identify salient features for each identified cluster. Categories with 75–99% representation were considered “highly likely,” those with 51–74% were deemed “likely,” and if no category exceeded 50%, it either indicated non-salience or appropriate category combination. Analyses were conducted using SPSS version 27.0 (IBM Corp., 2020).

## Results

### Validity and Reliability of the AFCCQ-IT

During the initial translation to Italian by two independent translators for the forward and two others for the backward translation, the research team made minor adjustments to ensure a consistent use of language in the final versions. The face validity testing involved five older individuals (age range 84 − 73, two males and three females), and the content validity testing included five academic experts doing research on ageing (*n* = 3), education (*n* = 1) and social policies (*n* = 1), who evaluated relevance of the items and their semantics. Older people found all items to be highly relevant (all items scored I-CVI = 1.0). Academic experts assessed item 2 “My house is accessible to the people who come to visit me” (I-CVI = 0.6) and item 6 “I find the range of events and activities sufficiently varied” (I-CVI = 0.4) less relevant or possibly confusing for older people. They were kept as items of the AFCCQ as older people indicated to have no problems with these two items. All other items were considered relevant (items 9, 13 and 16 I-CVI = 0.8; all remaining items I-CVI = 1.0).

Confirmatory Factor Analysis (CFA) for the original AFCCQ structure showed an excellent fit (χ^2^ = 2.329, CFI = 0.968, TLI = 0.959, RMSEA = 0.099, SRMR = 0.0283), also for the three cities (Table 3). All estimated covariance paths between factors were below 0.85, indicating sufficient discriminant validity and confirming that items measure distinct yet related factors. To validly compare the outcomes of the surveys in the three cities, MI was tested. Full scalar invariance was established without significant fit/model complexity trade-off compared to the metric model (scalar model: ΔCFI = 0.005; ΔRMSEA = 0.001; ΔSRMR = 0.0003). Thus, meaningful comparisons of the means or scores on the latent variable between groups, could now be made (Table 3).

**Table 3:** Fit of data from Italy, Verona, Venice and Palermo with the original model as described by Dikken et al., 2020, and level of measurement invariance between the three cities

	Model	Normed χ^2^ [DF]	Comparative Fit Index (CFI)	∆CFI	Tucker Lewis Index (TLI)	Root-Mean Squared Residual (SRMR)	∆ SRMR	Root-Mean Square Error of Approximation (RMSEA)	∆ RMSEA
**Italy**		**2.329 [194]**	**0.968**		**0.959**	**0.0283**		**0.033** **[0.029–0.037]**	
Verona		1.720 [194]	0.947		0.931	0.0415		0.042 [0.035–0.050]	
Venice		1.663 [194]	0.951		0.937	0.0400		0.040 [0.032–0.048]	
Palermo		1.380 (194)	0.972		0.964	0.0390		0.031 [0.021–0.039]	
MI	Configural	1.588 [582]	0.957	-	0.944	0.0415	-	0.022 [0.019–0.025]	-
	Metric	1.590 [610]	0.955	−0.002	0.943	0.0451	0.0036	0.022 [0.019–0.025]	0.000
	**Scalar**	**1.625 [638]**	**0.950**	**−0.005**	**0.940**	**0.0454**	**0.0003**	**0.023** **[0.020–0.025]**	**0.001**
	Residual Variance Invariance	1.835 [684]	0.928	−0.022	0.920	0.0507	0.0053	0.026 [0.024–0.029]	0.003

**Fig. 2:**
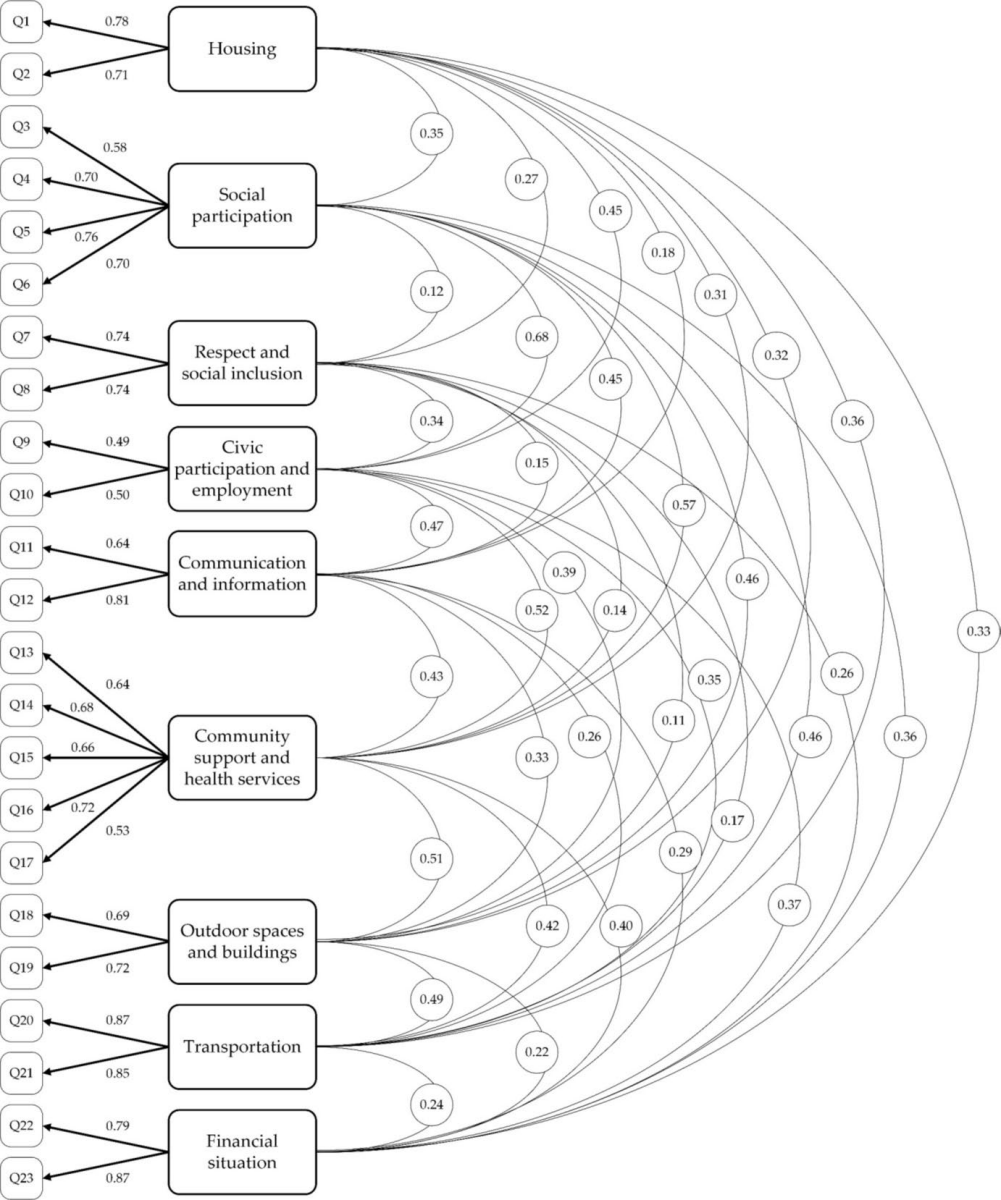
Model of the confirmatory factor analysis AFCCQ-IT

The evaluation of the internal consistency of the model derived from the final CFA (Fig. 2) involved examining the composite reliability (Table 4). The results revealed that most factors exceed the threshold of > 0.70, indicating a reasonable reliability. The domains of Communication and Information and Outdoor spaces and Buildings were at this threshold, with differences existing between cities. Notably, the domain of Civic Participation and Employment scored lower for the AFCCQ-IT, which could likely be attributed to the significant portion of people over the age of 65 in Italy, particularly women, who are not formally employed.

**Table 4:** Composite reliability per domain of the AFCCQ-IT

	Housing	Social Participation	Respect and Social Inclusion	Civic Participation and Employment	Communication and Information	Community Support and Health Services	Outdoor Spaces and Buildings	Transportation	Financial Situation
Italy	0.717	0.780	0.708	**0.390**	**0.696**	0.783	**0.667**	0.850	0.819
Verona	0.724	0.751	0.715	**0.413**	0.671	0.782	**0.628**	0.832	0.851
Venice	0.694	0.770	0.733	**0.378**	0.737	0.761	**0.620**	0.857	0.798
Palermo	0.743	0.781	0.712	**0.387**	0.715	0.772	0.723	0.840	0.819

### The Age-friendliness of the Italian Cities of Verona, Venice and Palermo

In general, the older population in the three cities in this study perceived the city’s age-friendliness as “neutral” (+) to “satisfied” (+++), depending on the domain. The overall score on the AFCCQ-IT was 13.32 ± 11.17, rated on a scale of −46 to + 46 (Table 5). An analysis of various AFCCQ-IT domains revealed that Italy attained a “neutral” rating in four out of nine domains. The domains of Respect and Social Inclusion and Housing achieved the highest scores (“satisfied”) with total scores of respectively 2.37 (Respect and Social Inclusion) and 2.30 (Housing) on a scale of −4 to + 4, reflected by positive scores across all three cities and districts (Appendix 1). Conversely, the lowest score (“slightly dissatisfied”) was observed in the domain of Community Support and Health Services in the city of Palermo, scoring − 0.33 on a scale of −4 to + 4, with negative scores present in five out of eight districts (Appendix 1). Overall, the city of Palermo scored lower in almost all domains compared to the Northern cities of Verona and Venice.

**Table 5:**
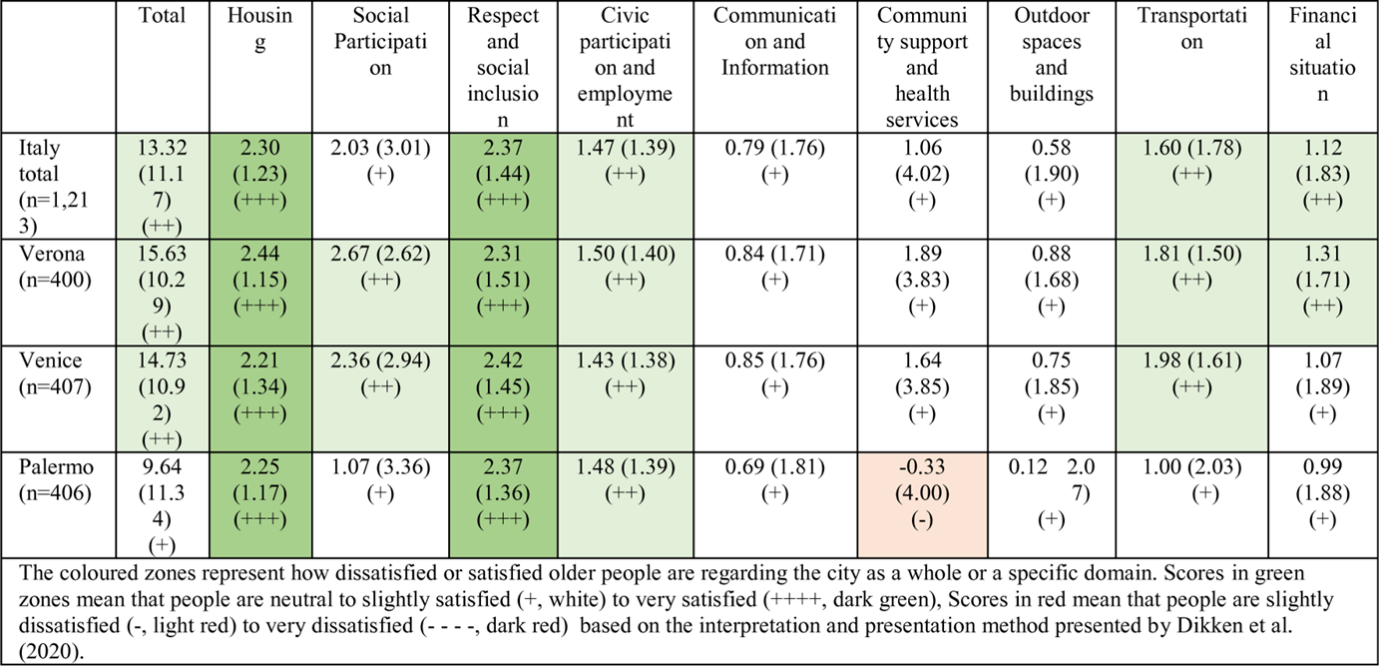
Scores (Mean +- SD) for AFCCQ domains for the cities of Verona, Venice and Palermo (n=number of participants)

### The Italian Age Friendly Typologies

**Fig. 3:**
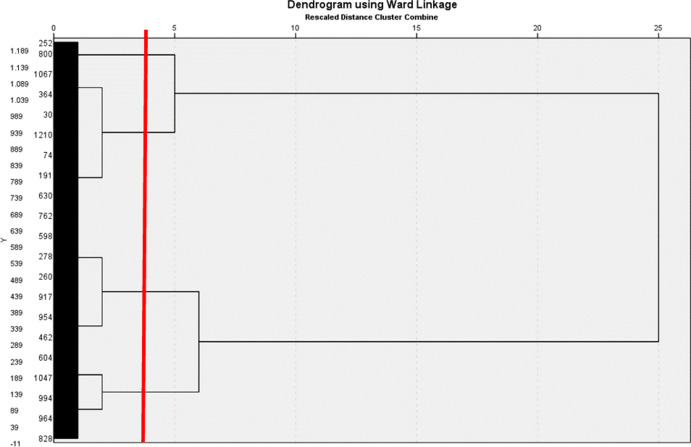
Dendogram resulting from the HCA

The dendrogram resulting from the HCA unveiled either two, four or seven distinct clusters (Fig. 3). After validation (split sample by city), the four-cluster solution proved to be the most stable.

Thereafter, *k*-means clustering was done revealing significant differences (< 0.001) among all four clusters across all nine domains of the AFCCQ (Table 6). Cluster 1, comprising 120 individuals, reported the least favourable levels of age-friendliness in Italy in all nine domains. Cluster 2 (381 individuals) reported negative scores for Community Support and Health Services (−1.39) and the domain of Transportation (−0.15). Cluster 3 was “the middle group”. Cluster 3 (531 individuals) scored relatively positive across all the domains of the AFCCQ. Cluster 4 (181 individuals) showed the most positive outcomes. While the domains of Housing and Respect and Social Inclusion showed significant differences, these differences were the least distinct among the clusters. Conversely, the remaining domains exhibited substantial disparities.

**Table 6:**
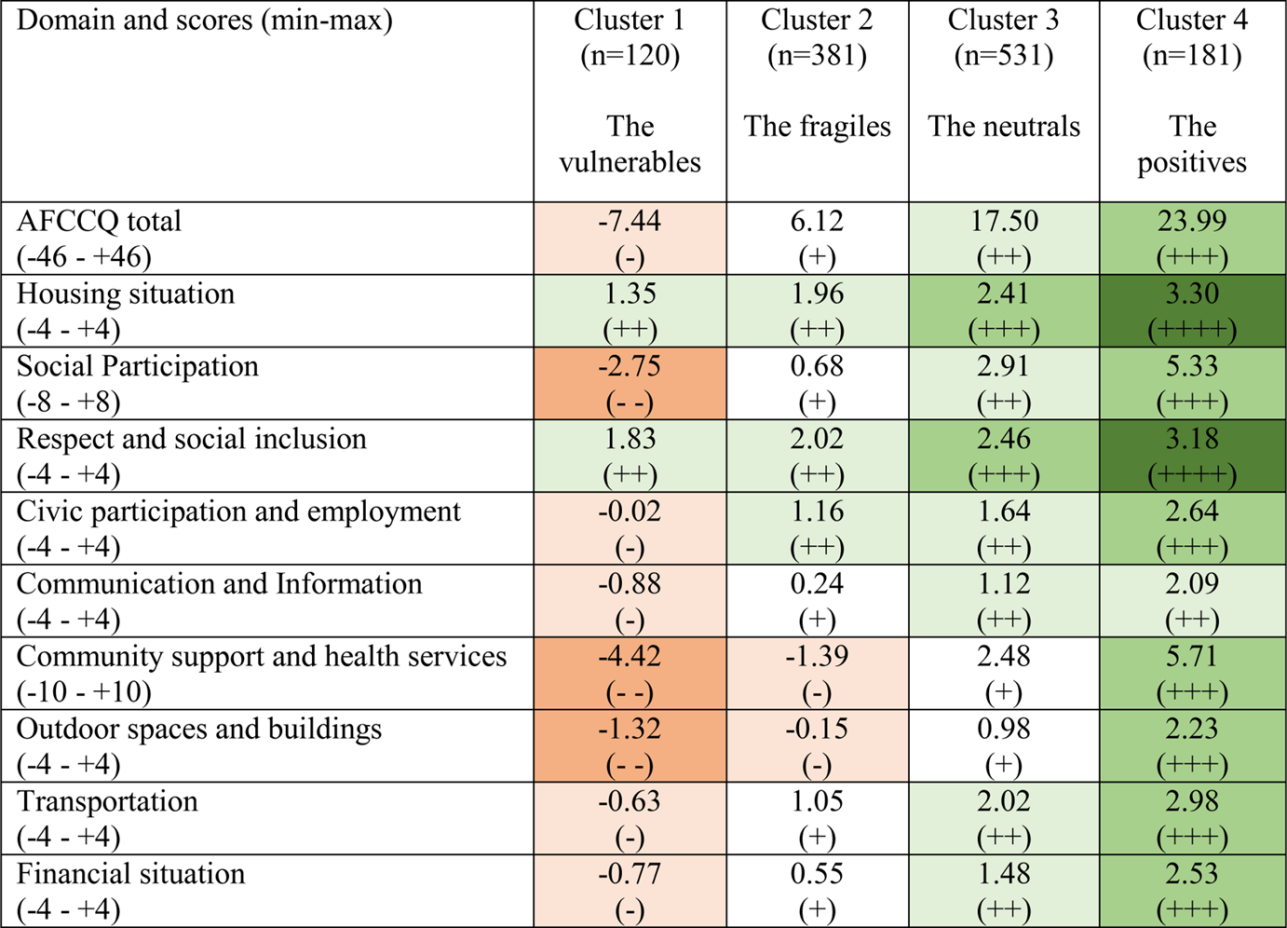
Cluster scores on AFCCQ domains

## Discussion

The present study delineates the process of adapting and validating the Age-Friendly Cities and Communities Questionnaire (AFCCQ) for the older population in Italy. It contributes to the further development and propagation of the World Health Organization’s initiative pertaining to age-friendly cities and communities, and provides a solution to the need for standardised and culturally-tailored instruments for assessing age-friendliness. Developed by Dikken et al. (2020) and included by the WHO (2020) as a promising practice for evaluating the perceived age-friendliness of cities and communities, the tool adopts a bottom-up methodology, transitioning from the perspectives of citizens to policymakers and local governance entities. This study details the phase of validating the AFCCQ-IT utilising data gathered from older adults residing in Venice, Verona, and Palermo, employing a sampling strategy that encompasses key socio-demographic factors and individuals from diverse districts within the three Italian municipalities. The newly adapted AFCCQ-IT exhibited adequate discriminant validity and demonstrated its efficacy as a robust instrument applicable within the Italian context. Data procured through employment of the AFCCQ-IT enabled the identification of four distinct clusters among older adults, each characterised by different urban experiences and perceptions of well-being. The four clusters are the expression of the different perceptions of the city’s age-friendliness, similar to what emerged from other researches which adopted the same framework (Dikken et al., 2020; Özer et al., 2022; van Hoof et al., 2022; van Hoof et al., 2024, Ivan et al., 2024; Yamada et al., 2023; Wasserman et al., 2025).

The description of the four clusters that emerged from our data, shows that older adults are a diversified group of citizens with different perceptions and expectations from their daily life and living environment (Table 7).

**Table 7:** The four age-friendly typologies based on socio-demographic data

	Cluster 1 The vulnerables (*n* = 120)	Cluster 2 The fragiles (*n* = 381)	Cluster 3 The neutrals (*n* = 531)	Cluster 4 The positives (*n* = 181)
City				
Verona n, (%)	22 (18.3%)	102 (26.8%)	205 (38.6%)	71 39.2%)
Venice n, (%)	29 (24.2%)	123 (32.3%)	183 (34.5%)	72 (39.8%)
Palermo n, (%)	69 (57.5%)	156 (40.9%)	143 (26.9%)	38 (21.0%)
Personal factors	75.8 ± 7.2 years old Likely to be female* (69.2%).	76.5 ± 7.5 years old Likely to be female* (69.2%).	76.3 ± 7.1 years old Likely to be female* (69.2%).	75.5 ± 7.4 years old Likely to be male* (55.2%).
Education level	Education is well distributed in the cluster	Education is well distributed in the cluster	Education is well distributed in the cluster	Likely to have **completed a higher level of education** ** (ISCED 5–8) (29.2%)
Housing situation	People are high likely to be owner-occupants** (86.7%). People who rented is 5.8% and 7.5% lived in social housing. Likely* to live with partner or other family members (62.5%).	People are high likely to be owner-occupants** (87.1%). People who rented is 7.3% and 5.5% lived in social housing. Likely* to live with partner or other family members (69.0%).	People are high likely to be owner-occupants** (92.1%). People who rented is 5.6% and 2.3% lived in social housing. Likely* to live with partner or other family members (70.6%).	People are high likely to be owner-occupants** (90.1%). People who rented is 8.3% and 1.7% lived in social housing. Likely* to live with partner or other family members (71.3%).
Health	Of this group, 35.0% of people mentioned having a chronic condition, 45.8% received some sort of healthcare and 14.2% used a mobility aid.	Of this group, 28.1% of people mentioned having a chronic condition, 38.1% received some sort of healthcare and 10.2% used a mobility aid.	Of this group, 30.5% of people mentioned having a chronic condition, 34.1% received some sort of healthcare and 4.5% used a mobility aid.	**Rather healthy compared to the other group**. Of this group, 24.9% of people mentioned having a chronic condition, 36.5% received some sort of healthcare and 2.8% used a mobility aid.
Quality of life	Mean score QoL 6.14 ± 2.2	Mean score QoL 6.91 ± 2.0	Mean score QoL 7.75 ± 1.7	**Higher mean score** QoL 8.38 ± 1.5
AFCCQ domain of interest	Low score (- -) for Social Participation, Community support and health services, Outdoor spaces and buildings. Dissatisfied (-) with Civic participation and employment, Communication and Information, Transportation, Financial situation.	Dissatisfied (-) with Community support and health services and Outdoor spaces and buildings.	Housing and Respect and Social inclusion are positively considered (+++)	**Good scores for Housing and Respect and Social inclusion** (++++)

**Highly likely *>* 75%, *Likely 51–74%, no salience *<* 50%

Cluster 1 – named the ‘vulnerables’ – is made up of 9.9% (n = 120) of the total number of respondents (n = 1.213) and consisted of people with a lower socio-economic status and a higher percentage of chronic conditions. They are concerned about their limited social participation and, above all, for the scarce availability of community support and health services. Excluding the domains of Housing and Respect and Social Inclusion, they rated all the other aspects of the city life negatively. This cluster is predominantly represented by older adults living in Palermo (57.5%), while in Venice the percentage is much lower, namely 24.2%, and 18.3% for Verona.

Cluster 2 – named the ‘fragiles’ – is made up of 31.4% of the respondents and shows fewer negative scores compared to Cluster 1. These negative scores are concentrated in the domains of Community Support and Health Services and Outdoor Spaces and Buildings. Palermo is again the city with higher percentages of respondents in this cluster (40.9%), while Venice’s percentage is 32.3% and Verona’s is 26.8%.

Cluster 3 – labelled the ‘neutrals’ – is made up of 43.7% of the respondents and their perception of the age-friendliness is both slightly positive (+) and positive (++). Housing and Respect and Social Inclusion are the domains with the higher scores. A significant difference from the Cluster 1 and Cluster 2 is the larger percentage of the older person living in the Northern cities (Verona 38.6%, Venice 34.5%), while for Palermo is only 26.9%. Cluster 4 – named the ‘positives’ – shows again the higher presence of older adults living in Verona (39.2%) and Venice (39.8%), while the score for Palermo is a mere 21.0%. This is the group with higher levels of education and who are relatively in better health compared to the other clusters. The perception of their quality of life (mean score 8.38 vs. 6.14 of Cluster 1) is another sign of the relative well-being of this group.

In order to better grasp the meaning of these data, the unresolved gap between the North and the South macro-areas of Italy must be recalled. The differences between the Italian North and South territories are rooted in the political and cultural history of the country, which predates the Italian unification in 1870. Despite the various economic and social development interventions that were programmed with ad hoc measures in the recent past, there is still a substantial and visible gap between the country’s different geographical areas. The northern regions of Italy are among the most developed areas in Europe, whilst in contrast the southern regions are among the least developed in Europe (Bertani, 2015). The analysis of a wide range of indicators highlights a constant negative differential of the geographical areas of the South with respect to the Centre of the country and the North. Economic indicators such as GDP per capita, the poverty index and the unemployment rates, demographic indicators such as the fertility rates, and socio-cultural indicators such as the educational performance highlight a distance between North and South that still appears difficult to realign. When citing these figures, one must bear in mind that the southern regions have the highest rates of underground economy and irregular work in the European Union (ISTAT 2023b). The geographical differential for these indicators between the North and South emerges in similar research. For example, the ‘Report on the Quality of Life in European Cities’ (European Commission, 2023) presents the experiences and opinions of city dwellers across Europe, covering 83 cities across the continent. Palermo and Verona were included, and data show that the percentages of residents who were satisfied with the city where they live were 89% in Verona and 62% in Palermo, a difference of 27% points (European Commission, 2023, page 14).

The four clusters emerging from the data analysis suggest summarising a policy perspective in terms of potential interventions (Table 8). Cluster 1 (the vulnerables) needs significant and rapid forms of help and support to deal with the stresses and the weaknesses that characterise this group of older people. Being the cluster which is the most socially isolated and which faces the greatest health problems, it is in need of an articulated intervention that sees the supporting actions of both institutional bodies (municipalities, social services, health services) and the local network of voluntary associations to facilitate pathways to social inclusion and personalised healthcare interventions. Cluster 2 (the fragiles) is slightly less problematic than Cluster 1. This cluster could benefit from similar actions, even in a more simplified manner and with a particular focus on Community Support and Health Services.

**Table 8:** Potential policy recommendations for each of the four clusters

Cluster 1 The vulnerables	Cluster 2 The fragiles	Cluster 3 The neutrals	Cluster 4 The positives
This cluster will benefit from relevant policies co-designed by public bodies and voluntary associations active in the cities neighbours in order to improve: - Social connectedness - Community support - Healthcare services	This cluster will benefit from policies designed by public bodies in order to improve: - Community support - Healthcare services	Policies designed for continuation and stimulation of positive perceptions	This cluster is in a condition where policies are not strictly necessary

The perception on age-friendliness of Cluster 3 (the neutrals) is fairly good. Relevant policies seem unnecessary, even though age-friendly policies could be essential to maintain or enhance this condition. Cluster 4 (the positives) is in such a condition that policies are not strictly necessary, but the older adults constituting this profile could be encouraged to play the role of ‘positive example’ for the engagement of older adults with the profiles presented in Cluster 1 ‘the vulnerable’ in community empowerment projects and activities. Especially for the first two clusters, the most isolated and vulnerable older adults, the three Italian cities involved in this research could follow and adapt existing promising practices to their socio-cultural local context. For instance, the City of Ottawa (Canada) launched a programme for engaging the broader community named ‘Community Connect’, which promotes the awareness and identification of vulnerable older adults and provides telephone information and referral services (Ottawa Public Health, 2013). In a similar vein, a specific programme for isolated older adults is supported by the City of Saint-Etienne (France) to combat the isolation of older adults by promoting social ties and civic involvement (World Health Organization, 2018). The main objective is to break down isolation by offering non-professional activities such as social visits (board games, discussions, etc.), regular telephone calls as well as (cultural) outings or walks.

## Conclusions

This study presents the translation, cultural adaptation and validation of the AFCCQ-IT, an established standardised and globally validated tool, for assessing the perceived age-friendliness among Italy’s older population. The adapted instrument demonstrated robustness, aligning with the WHO’s eight dimensions for age-friendly cities. The research illustrates how the AFCCQ-IT can delineate distinct typologies (clusters) of older adults and their different experiences within Northern (Venice, Verona) and Southern (Palermo) Italian cities. To this end, policymakers - both at the national and local level - need to be equipped with the necessary tools to monitor and promote age-friendly environments and their effects on the older population. The AFCCQ-IT can be used in the Italian context and allows stakeholders to deal with the sub-national diversity that characterises Italy, ensuring that progress towards more age-friendly environments is equitable across the country.

## Electronic supplementary material

Below is the link to the electronic supplementary material.

Supplementary Material 1

## Figures and Tables

**Table 9:**
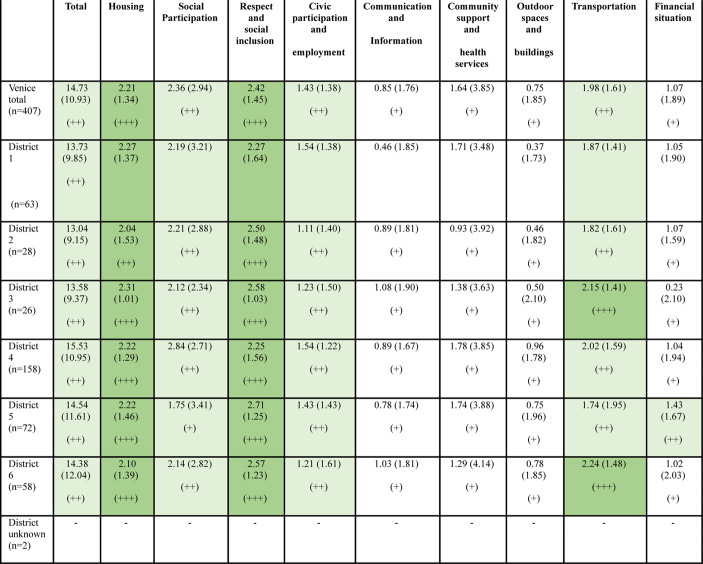
Scores (Mean +- SD) for AFCCQ domains for the municipality of Venice and its districts* (*n*=number of participants)

The coloured zones represent how dissatisfied or satisfied older people are regarding the city as a whole or a specific domain. Scores in green zones mean that people are neutral to slightly satisfied (+, white) to very satisfied (++++, dark green), Scores in red mean that people are slightly dissatisfied (-, light red) to very dissatisfied (- - - -, dark red) based on the interpretation and presentation method presented by Dikken et al., 2020. *District 1: Old city, Murano, Burano (islands) ; District 2: Lido-Pellestrina (islands); District 3: Favaro-Veneto (mainland) ; District 4: Mestre-Carpenedo (mainland); District 5: Chirignago-Zelarino (mainland); District 6: Marghera (mainland)

**Table 10:**
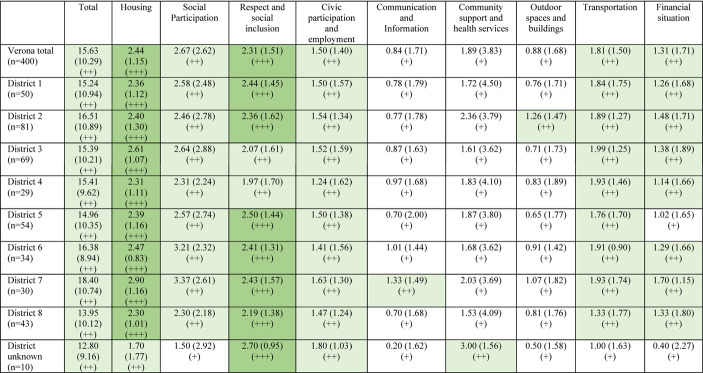
Scores (Mean +- SD) for AFCCQ domains for the municipality of Verona and its districts* (*n*=number of participants)

The coloured zones represent how dissatisfied or satisfied older people are regarding the city as a whole or a specific domain. Scores in green zones mean that people are neutral to slightly satisfied (+, white) to very satisfied (++++, dark green), Scores in red mean that people are slightly dissatisfied (-, light red) to very dissatisfied (- - - -, dark red) based on the interpretation and presentation method presented by Dikken et al., 2020. *District 1: Old City; District 2: North-West; District 3 West; District 4: South-West; District 5: South; District 6: East; District 7: South-East; District 8: North-East

**Table 11:**
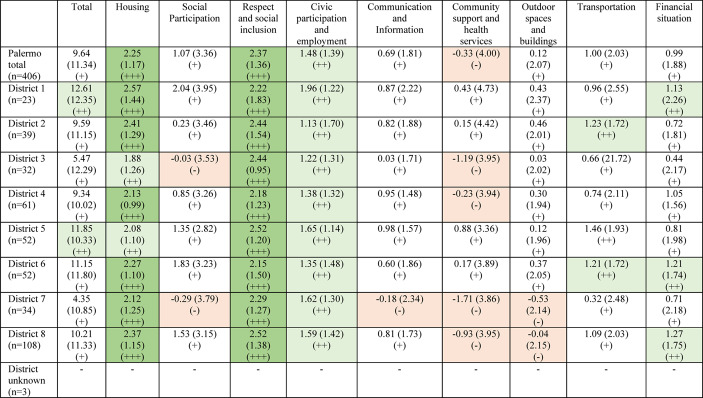
Scores (Mean +- SD) for AFCCQ domains for the municipality of Palermo and its districts* (*n*=number of participants)

The coloured zones represent how dissatisfied or satisfied older people are regarding the city as a whole or a specific domain. Scores in green zones mean that people are neutral to slightly satisfied (+, white) to very satisfied (++++, dark green), Scores in red mean that people are slightly dissatisfied (-, light red) to very dissatisfied (- - - -, dark red) based on the interpretation and presentation method presented by Dikken et al., 2020. *District 1: Tribunali-Castellammare, Palazzo Reale - Monte di Pietà; District 2: Oreto- Stazione (Parte), Settecannoli, Brancaccio-Ciaculli; District 3: Oreto- Stazione (Parte), Villagrazia-Falsomiele; District 4: Montegrappa - S. Rosalia, Cuba-Calatafimi, Mezzomonreale -Villa Tasca, Altarello, Boccadifalco; District 5: Zisa, Noce, Uditore-Passo Di Rigano, Borgo Nuovo; District 6: Cruillas - S.Giovanni Apostolo, Resuttana - S.Lorenzo; District 7: Tommaso Natale - Sferracavallo, Partanna Mondello, Pallavicino, Arenella - Vergine Maria; District 8: Malaspina - Palagonia, Libertà, Politeama, Montepellegrino

## Data Availability

This study uses data from the Age-Friendly Cities and Communities Questionnaire survey (AFCCQ - Italian version), designed and implemented under the Programme Age-It (https://ageit.eu/wp/en/), Work package 3.1 – Spoke 10 “Mainstreaming ageing by building institutional mechanisms for better and future-oriented health policy making and prevention”. The original dataset can be downloaded from the open repository Zenodo DOI 10.5281/zenodo.11084903.
