# Supplementary material for: Getting Older People’s Voices Heard: A Quantitative Study Using the Validated Italian Age-Friendly Cities and Communities Questionnaire in Venice, Verona and Palermo
Source: J Cross Cult Gerontol. 2025 May 14;40(2):209–34. doi: 10.1007/s10823-025-09532-1 (PMC12137376; doi:10.1007/s10823-025-09532-1)
Supplement: Supplementary file 1 — Supplementary Material 1 [file 10823_2025_9532_MOESM1_ESM.docx]

The Age-Friendly Cities and Communities Questionnaire AFCCQ (Italian)

| **Item** |  | **Domain** |  | **Totally disagree/ Totalmente disaccordo** |  | **Disagree/ In disaccordo** |  | **Neutral/ Ne accordo, ne disaccordo** |  | **Agree/ D’accordo** |  | **Totally agree/ Totalmente d’accordo** |
| --- | --- | --- | --- | --- | --- | --- | --- | --- | --- | --- | --- | --- |
|  |  | **HOUSING / ABITAZIONE** |  |  |  |  |  |  |  |  |  |  |
| Q1 |  | My house is accessible to me / La mia casa si trova in una zona o in un edificio a me facilmente accessibile |  |  |  |  |  |  |  |  |  |  |
| Q2 |  | My house is accessible to the people who come to visit me / La mia casa si trova in una zona o in un edificio facilmente accessibile alle persone che vengono a farmi visita |  |  |  |  |  |  |  |  |  |  |
|  |  | **SOCIAL PARTICIPATION / SOCIALITA’** |  |  |  |  |  |  |  |  |  |  |
| Q3 |  | There are enough opportunities to meet people in my neighbourhood / Nel mio quartiere/zona ci sono sufficienti occasioni per incontrare altre persone |  |  |  |  |  |  |  |  |  |  |
| Q4 |  | Activities and events are organised in places that are accessible to me / Attività ed eventi sono organizzati in luoghi che sono per me accessibili |  |  |  |  |  |  |  |  |  |  |
| Q5 |  | The information about activities and events is enough for me and also suitable for me Le informazioni sulle attività ed eventi sono sufficienti e adatte a me |  |  |  |  |  |  |  |  |  |  |
| Q6 |  | I find the range of events and activities sufficiently varied / L’offerta di eventi e attività è sufficientemente varia |  |  |  |  |  |  |  |  |  |  |
|  |  | **RESPECT AND SOCIAL INCLUSION / RISPETTO E INCLUSIONE SOCIALE** |  |  |  |  |  |  |  |  |  |  |
| Q7 |  | I sometimes get annoying or negative remarks because of my age / Qualche volta ricevo commenti fastidiosi o negativi a causa della mia età |  |  |  |  |  |  |  |  |  |  |
| Q8 |  | I sometimes face discrimination because of my age / Qualche volta vengo discriminato a causa della mia età |  |  |  |  |  |  |  |  |  |  |
|  |  | **CIVIC PARTICIPATION AND EMPLOYMENT / VITA NELLA COMUNITÀ** |  |  |  |  |  |  |  |  |  |  |
| Q9 |  | I have enough opportunities to interact with younger generations / Ho abbastanza occasioni di incontrare persone più giovani |  |  |  |  |  |  |  |  |  |  |
| Q10 |  | I feel like a valued member of society/  Nella mia comunità mi sento una persona valorizzata |  |  |  |  |  |  |  |  |  |  |
|  |  | **COMMUNICATION AND INFORMATION / COMUNICAZIONE E INFORMAZIONI** |  |  |  |  |  |  |  |  |  |  |
| Q11 |  | Printed and digital information from the municipality and other social institutions is easy to read in terms of font and size / Le informazioni che ricevo, stampate o su Internet, dal Comune o da altre istituzioni sono facili da leggere (nel senso che le lettere sono grandi e facili da leggere) |  |  |  |  |  |  |  |  |  |  |
|  |  |  |  |  |  |  |  |  |  |  |  |  |
| Q12 |  | Printed and digital information from the municipality and other social institutions is written in understandable language / Le informazioni che ricevo, stampate o su Internet, dal Comune o da altre istituzioni sono facili da comprendere (nel senso che sono in un linguaggio comprensibile) |  |  |  |  |  |  |  |  |  |  |
|  |  |  |  |  |  |  |  |  |  |  |  |  |
|  |  | **COMMUNITY SUPPORT AND HEALTH SERVICES / SERVIZI SANITARI E DI SOSTEGNO DELLA COMUNITÀ** |  |  |  |  |  |  |  |  |  |  |
| Q13 |  | The supply of care and welfare in my city is enough for me / Nella mia città l'offerta di servizi sanitari e di servizi di assistenza è sufficiente |  |  |  |  |  |  |  |  |  |  |
| Q14 |  | When I am ill, I receive the care and help I need / Quando sono ammalato/a, ricevo tutte le cure del servizio sanitario di cui ho bisogno |  |  |  |  |  |  |  |  |  |  |
| Q15 |  | If necessary, I can easily reach care and welfare services by telephone and in person / In caso di necessità, posso facilmente raggiungere i servizi sanitari (medico, distretto, ospedale) e di assistenza sociale sia telefonicamente che di persona |  |  |  |  |  |  |  |  |  |  |
| Q16 |  | I have enough information about care and welfare services in my neighbourhood / Ho sufficienti informazioni sui servizi sanitari e di assistenza sociale presenti nel mio quartiere/zona |  |  |  |  |  |  |  |  |  |  |
| Q17 |  | Care and welfare workers in my neighbourhood are sufficiently respectful / Nel mio quartiere/zona gli operatori sanitari e assistenziali sono sufficientemente rispettosi |  |  |  |  |  |  |  |  |  |  |
|  |  | **OUTDOOR SPACES AND BUILDINGS / SPAZI APERTI E BARRIERE ARCHITETTONICHE** |  |  |  |  |  |  |  |  |  |  |
| Q18 |  | My neighbourhood is sufficiently accessible for a wheeled walker or wheelchair / Il mio quartiere/zona è sufficientemente accessibile per una persona in sedia a rotelle o che usa il deambulatore/bastone |  |  |  |  |  |  |  |  |  |  |
| Q19 |  | The shops in my neighbourhood are sufficiently accessible with a wheeled walker or wheelchair/ Nel mio quartiere/zona i negozi sono sufficientemente accessibili per una persona in sedia a rotelle o che usa il deambulatore/bastone |  |  |  |  |  |  |  |  |  |  |
|  |  | **TRANSPORTATION / TRASPORTI PUBBLICI** |  |  |  |  |  |  |  |  |  |  |
| Q20 |  | I can easily get on the bus or tram in my neighbourhood / Nel mio quartiere/zona posso prendere facilmente i mezzi pubblici |  |  |  |  |  |  |  |  |  |  |
| Q21 |  | The bus and tram stops in my neighbourhood are easy to reach and use / Nel mio quartiere/zona le fermate dei mezzi pubblici sono facilmente raggiungibili e adatte agli spostamenti |  |  |  |  |  |  |  |  |  |  |
|  |  | **FINANCIAL SITUATION / SITUAZIONE ECONOMICA** |  |  |  |  |  |  |  |  |  |  |
| Q22 |  | My income is sufficient to cover my basic needs without any problems / Il mio reddito è sufficiente per coprire i bisogni più importanti |  |  |  |  |  |  |  |  |  |  |
| Q23 |  | I live well on my income / Il mio reddito mi consente di vivere bene |  |  |  |  |  |  |  |  |  |  |
